# Simultaneous Antibiofilm and Antiviral Activities of an Engineered Antimicrobial Peptide during Virus-Bacterium Coinfection

**DOI:** 10.1128/mSphere.00083-16

**Published:** 2016-05-04

**Authors:** Jeffrey A. Melvin, Lauren P. Lashua, Megan R. Kiedrowski, Guanyi Yang, Berthony Deslouches, Ronald C. Montelaro, Jennifer M. Bomberger

**Affiliations:** a Department of Microbiology and Molecular Genetics, University of Pittsburgh, Pittsburgh, Pennsylvania, USA; b Tsinghua University School of Medicine, Beijing, China; c Center for Vaccine Research, University of Pittsburgh, Pittsburgh, Pennsylvania, USA; University of Kentucky

**Keywords:** antimicrobial agents, antiviral agents, biofilms

## Abstract

Antimicrobial-resistant infections are an urgent public health threat, and development of novel antimicrobial therapies has been painstakingly slow. Polymicrobial infections are increasingly recognized as a significant source of severe disease and also contribute to reduced susceptibility to antimicrobials. Chronic infections also are characterized by their ability to resist clearance, which is commonly linked to the development of biofilms that are notorious for antimicrobial resistance. The use of engineered cationic antimicrobial peptides (eCAPs) is attractive due to the slow development of resistance to these fast-acting antimicrobials and their ability to kill multidrug-resistant clinical isolates, key elements for the success of novel antimicrobial agents. Here, we tested the ability of an eCAP, WLBU2, to disrupt recalcitrant *Pseudomonas aeruginosa* biofilms. WLBU2 was capable of significantly reducing biomass and viability of *P. aeruginosa* biofilms formed on airway epithelium and maintained activity during viral coinfection, a condition that confers extraordinary levels of antibiotic resistance. Biofilm disruption was achieved in short treatment times by permeabilization of bacterial membranes. Additionally, we observed simultaneous reduction of infectivity of the viral pathogen respiratory syncytial virus (RSV). WLBU2 is notable for its ability to maintain activity across a broad range of physiological conditions and showed negligible toxicity toward the airway epithelium, expanding its potential applications as an antimicrobial therapeutic.

**IMPORTANCE** Antimicrobial-resistant infections are an urgent public health threat, making development of novel antimicrobials able to effectively treat these infections extremely important. Chronic and polymicrobial infections further complicate antimicrobial therapy, often through the development of microbial biofilms. Here, we describe the ability of an engineered antimicrobial peptide to disrupt biofilms formed by the ESKAPE (*Enterococcus faecium*, *Staphylococcus aureus*, *Klebsiella pneumoniae*, *Acinetobacter baumannii*, *Pseudomonas aeruginosa*, and *Enterobacter* species) pathogen *Pseudomonas aeruginosa* during coinfection with respiratory syncytial virus. We also observed antiviral activity, indicating the ability of engineered antimicrobial peptides to act as cross-kingdom single-molecule combination therapies.

## Abstract (precis)

Antimicrobial-resistant infections are an urgent public health threat, making development of novel antimicrobials able to effectively treat these infections extremely important. Chronic and polymicrobial infections further complicate antimicrobial therapy, often through the development of microbial biofilms. Here, we describe the ability of an engineered antimicrobial peptide to disrupt biofilms formed by the ESKAPE (*Enterococcus faecium*, *Staphylococcus aureus*, *Klebsiella pneumoniae*, *Acinetobacter baumannii*, *Pseudomonas aeruginosa*, and *Enterobacter* species) pathogen *Pseudomonas aeruginosa* during coinfection with respiratory syncytial virus. We also observed antiviral activity, indicating the ability of engineered antimicrobial peptides to act as cross-kingdom single-molecule combination therapies.

## INTRODUCTION

Respiratory bacterial infections are increasingly complicated by the inability of antibiotic therapy to eradicate the offending pathogen, in both acute respiratory diseases such as community-acquired pneumonia (CAP), hospital-acquired pneumonia (HAP), and ventilator-acquired pneumonia (VAP) (1) and chronic respiratory diseases such as cystic fibrosis (CF), chronic obstructive pulmonary disorder (COPD), and the common respiratory infection complication chronic or recurrent otitis media (OM) (2, 3). Chronic infections are often characterized by development of bacterial biofilms, a mode of growth that confers greatly increased antimicrobial resistance (4) and which may be potentiated by polymicrobial interactions (3, 5). The difficulty in treating these infections is compounded by the slow development of novel antimicrobials and the rapid development of resistance to newly deployed antibiotics (6).

CF is a deadly genetic disease arising from defects in ion transport by the mucosal epithelium. The primary source of morbidity and mortality in CF patients is chronic pulmonary infection, with *Pseudomonas aeruginosa* being particularly correlated with decline in quality of life and life expectancy (7). *P. aeruginosa* is a member of the ESKAPE pathogens, including *Enterococcus faecium*, *Staphylococcus aureus*, *Klebsiella pneumoniae*, *Acinetobacter baumannii*, *P. aeruginosa*, and *Enterobacter* species, for its ability to rapidly acquire resistance to antibiotics and its role as a major nosocomial pathogen (8). Respiratory virus infection, particularly due to respiratory syncytial virus (RSV), is also a significant cause of morbidity and is associated with *P. aeruginosa* infection (9). Our recent study revealed respiratory virus coinfection induced the rapid transition of *P. aeruginosa* to a biofilm mode of growth to colonize the airway epithelium (5). Bacteria in biofilms are notoriously recalcitrant to killing by antimicrobials and immune effectors (4), especially under the conditions encountered in the CF lung, where thickened mucus secretions and altered ion concentrations further inhibit bacterial clearance mechanisms (10). Polymicrobial infections are common in chronic diseases like CF and can further exacerbate the inability of antimicrobial therapy to clear infections (11).

**FIG 1:**
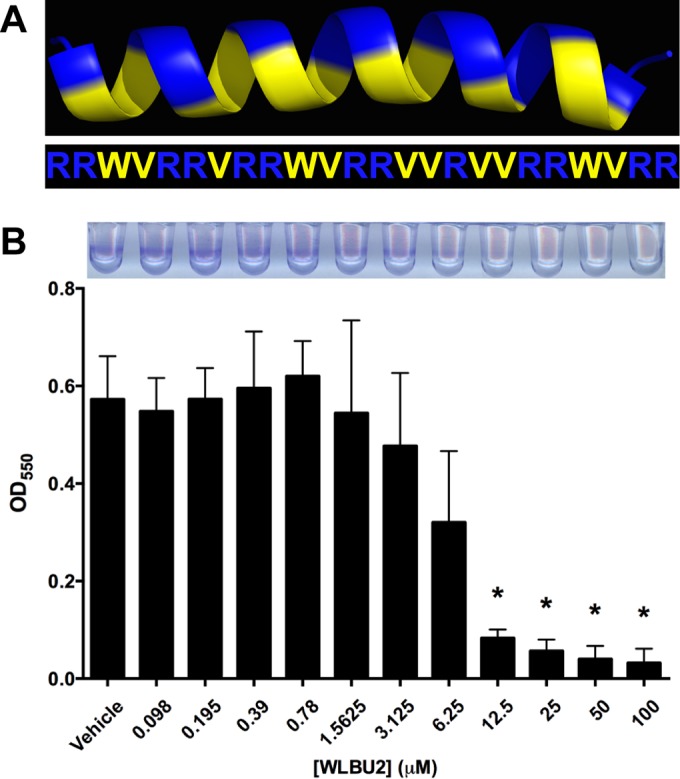
WLBU2 disrupts bacterial biofilms formed on abiotic surfaces. (A) Structural model and amino acid sequence of WLBU2 revealing the amphipathic nature of the helix (32, 33). Basic, positively charged residues are in blue, and hydrophobic residues are in yellow. (B) *P. aeruginosa* biofilms were grown statically for 24 h on vinyl microtiter plates prior to 1 h of treatment with various concentrations of WLBU2. Biomass was quantified via crystal violet staining, solubilization, and measurement of absorbance at 550 nm. A representative staining pattern is shown above. Data are means from three independent experiments. Error bars indicate standard deviations. *, *P* < 0.05 (compared to vehicle).

Antimicrobial peptides are gaining increasing interest as potential therapeutics due to their ability to kill antibiotic-resistant bacteria (12) and their potential as antibiofilm agents (13). However, results with diverse natural antimicrobial peptides, also known as cationic host defense peptides, have revealed intrinsic limitations to their use in treatment of human infectious diseases. We previously reported the engineered cationic antimicrobial peptide (eCAP) WLBU2, a 24-residue peptide composed of only arginine, valine, and tryptophan rationally designed to optimize amphipathic helical structure (Fig. 1A), maximize antibacterial membrane interactions, and minimize potential cytotoxicity toward the host (14). WLBU2 maintains activity under complex biological conditions such as in blood, serum, and murine models of acute *P. aeruginosa* infection (15–17). Importantly, WLBU2 demonstrates activity against the most common multidrug-resistant (MDR) and extensively drug resistant (XDR) pathogens, including bacterial isolates from CF patients (18). Since WLBU2 is highly effective against *P. aeruginosa* in planktonic culture (14, 15, 18, 19), the goal of this study was to examine if WLBU2 had antibiofilm activity for *P. aeruginosa*, particularly in the setting of incredibly antibiotic-resistant biofilms formed during virus-bacterium coinfection.

## RESULTS AND DISCUSSION

### WLBU2 disrupts *P. aeruginosa* biofilms via membrane disruption.

**FIG 2:**
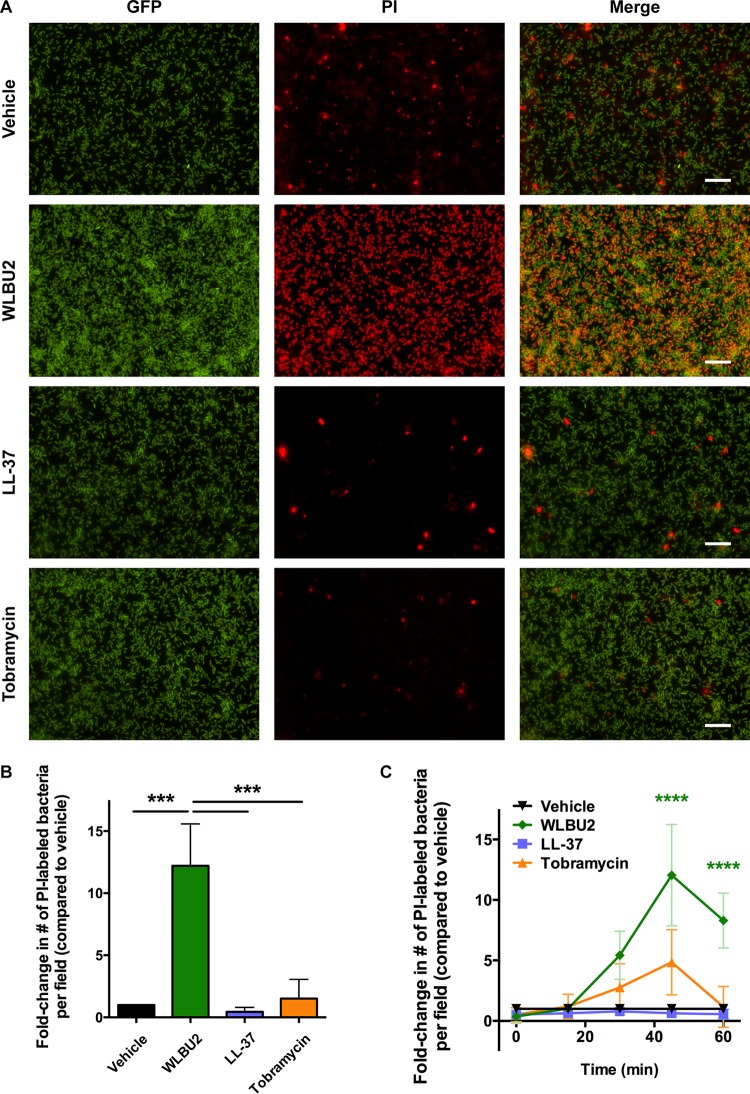
WLBU2 disrupts bacterial membranes. (A) Representative fluorescence microscopy images of GFP-producing *P. aeruginosa* cells grown on glass for 24 h prior to treatment with 50 µM WLBU2, 50 µM LL-37, 1,000 µg/ml tobramycin, or vehicle for 1 h. Bacteria were simultaneously exposed to propidium iodide (PI) during treatment. Scale bars, 50 µm. (B) Quantification of PI-stained cells in panel A, expressed as fold change in number of PI-stained cells compared to vehicle treatment. (C) Bacteria grown and treated as in panel A were tracked over time for the number of propidium iodide-positive cells, with significance reported compared to all other treatments. Data are means from three independent experiments. Error bars indicate standard deviations. ***, *P* < 0.001; ****, *P* < 0.0001.

We first examined the ability of WLBU2 to disrupt *P. aeruginosa* biofilms grown on abiotic surfaces, which confers a 10- to 100-fold increase in antibiotic resistance (20). *P. aeruginosa* biofilms were grown on plastic and treated for 1 h with increasing concentrations of WLBU2 before quantification by crystal violet staining. *P. aeruginosa* biofilms were readily disrupted by WLBU2 in a dose-dependent fashion, with approximately 90% biomass reduction above 10 µM (Fig. 1B). WLBU2 was designed to optimally interact with bacterial membranes (14) and has been shown to strongly interact with lipopolysaccharide (21). Accordingly, treatment of *P. aeruginosa* cells grown on glass with WLBU2 resulted in rapid uptake of propidium iodide (PI) (~10-fold greater than treatment with vehicle), while treatment with tobramycin or LL-37 had negligible effects on propidium iodide uptake (Fig. 2). These results suggest that WLBU2 acts similarly to polymyxins and other membrane-targeting antibiotics in targeting bacterial membranes (22).

### WLBU2 disrupts *P. aeruginosa* biofilms formed on airway epithelium.

**FIG 3:**
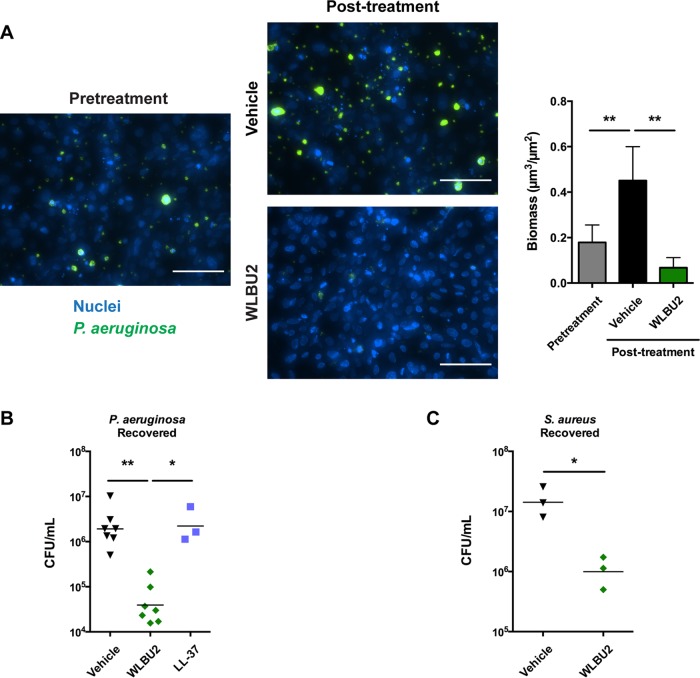
WLBU2 disrupts bacterial biofilms grown on bronchial epithelial cells. (A) Representative images and biomass quantification of *P. aeruginosa* biofilms during growth on bronchial epithelial cells before and after 1 h of treatment with 10 µM WLBU2. Bronchial epithelial cell nuclei are in blue, and *P. aeruginosa* cells are in green. Scale bars, 100 µm. (B) *P. aeruginosa* cells recovered from biofilms grown on bronchial epithelial cells after 1 h of treatment with 50 µM WLBU2 or LL-37. (C) *S. aureus* cells recovered from biofilms grown on bronchial epithelial cells after 2 h of treatment with 50 µM WLBU2. Data are means from three or more independent experiments. Error bars indicate standard deviations. *, *P* < 0.05; **, *P* < 0.01.

Next, we examined the disruption of *P. aeruginosa* biofilms grown on airway epithelial cells, a condition that confers a further 10- to 100-fold increase in antibiotic resistance (23). Bronchial epithelial cells were grown as a confluent monolayer, and nuclei were stained. The cells were inoculated with green fluorescent protein (GFP)-producing *P. aeruginosa*, and biofilms were allowed to form under perfusion prior to treatment with WLBU2 for 1 h and evaluated by fluorescence microscopy. We visualized biofilm biomass reduction by approximately 85% compared to treatment with vehicle (Fig. 3A). We also used a static coculture model (5), where bronchial epithelial cells were first polarized at the air-liquid interface. Bacteria were then allowed to attach and form mature biofilms, which display commonly observed biofilm characteristics such as production of extracellular matrix, expression of biofilm-associated genes, requirement of essential biofilm genes, and induction of quorum sensing. Importantly, these biofilms are extremely resistant to antibiotic clearance, with peak lung treatment concentrations of tobramycin, ciprofloxacin, or imipenem failing to eradicate them even after long treatment times (23, 24). Mature biofilms were treated with antimicrobial peptides for 1 h, and the remaining viable bacteria were quantified. We again observed *P. aeruginosa* biofilm disruption with WLBU2 treatment, reducing CFU by approximately 50-fold compared to treatment with vehicle (Fig. 3B). Of note, the natural mucosal antimicrobial peptide LL-37 was completely ineffective under similar conditions. Transepithelial electrical resistance (TEER) and cell viability were not affected by the doses of peptide or during the exposure times used in these experiments (see Fig. S1 in the supplemental material), demonstrating that epithelial tight junctions remained intact and that WLBU2 exhibited negligible cytotoxicity, a feature of WLBU2 that has been previously observed *in vitro* (14, 19) and *in vivo* (15, 16). Additionally, we have previously shown that the infections utilized do not cause overt cytotoxicity in the time frame and multiplicity of infection (MOI) used in our coculture model (5). WLBU2 has also been shown to be highly effective *in vitro* against *Staphylococcus aureus* (14, 18), another major pathogen in CF (25). Using our static coculture model, WLBU2 disrupted biofilms established by methicillin-resistant *S. aureus* (MRSA) on polarized bronchial epithelial cells, reducing CFU by approximately 15-fold (Fig. 3C).

10.1128/mSphere.00083-16.1 Figure S1  WLBU2 does not alter integrity of airway epithelium during treatment. (A) Transepithelial electrical resistance (TEER) was measured for bronchial epithelial cells during apical treatment with 10 or 50 µM antimicrobial peptide. (B) Lactate dehydrogenase release by polarized bronchial epithelial cells after apical treatment with 20 or 50 µM WLBU2. Data are means from three independent experiments. Error bars indicate standard deviations. Download Figure S1, TIF file, 0.3 MB. Copyright © 2016 Melvin et al. 2016 Melvin et al. This content is distributed under the terms of the Creative Commons Attribution 4.0 International license.

### WLBU2 disrupts *P. aeruginosa* biofilms formed on airway epithelium during RSV coinfection.

**FIG 4:**
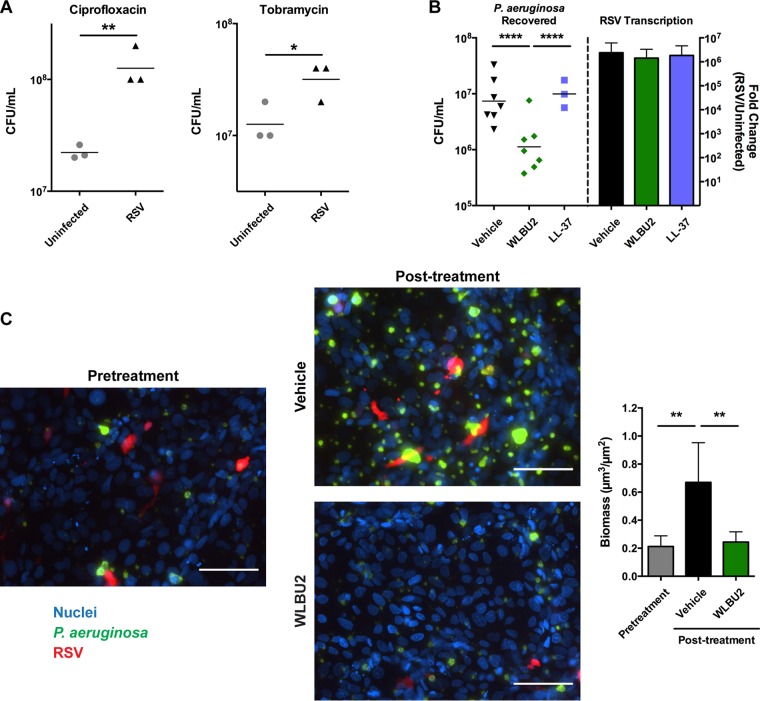
WLBU2 disrupts *P. aeruginosa* biofilms during RSV coinfection of bronchial epithelial cells. (A) *P. aeruginosa* cells recovered from biofilms grown on bronchial epithelial cells, either uninfected or infected with RSV, after 1.5 h of treatment with 500 ng/ml ciprofloxacin or 1 mg/ml tobramycin. (B) *P. aeruginosa* cells recovered from biofilms grown on RSV-infected bronchial epithelial cells after 1 h of treatment with 50 µM WLBU2 or LL-37 and viral replication in the epithelial cells. Bronchial epithelial cell nuclei are in blue, *P. aeruginosa* cells are in green, and RSV is in red. Scale bars, 100 µm. (C) Representative images and biomass quantification of *P. aeruginosa* biofilms during growth on RSV-infected bronchial epithelial cells before and after 1 h of treatment with 10 µM WLBU2. Data are means from three or more independent experiments. Error bars indicate standard deviations. *, *P* < 0.05; **, *P* < 0.01; ****, *P* < 0.0001.

Respiratory viral infection is correlated with *P. aeruginosa* infection in CF patients (9), and polymicrobial infections can decrease antimicrobial susceptibility (11). Accordingly, *P. aeruginosa* biofilms formed on polarized bronchial epithelial cells were less susceptible to peak concentrations of two antibiotics commonly used to treat chronic *P. aeruginosa* infections in CF patients, ciprofloxacin and tobramycin, during RSV coinfection (Fig. 4A). To determine the efficacy of WLBU2 during an established virus-bacterium coinfection, we investigated its ability to disrupt *P. aeruginosa* biofilms formed on polarized bronchial epithelial cells during RSV coinfection in our static coculture model. Polarized bronchial epithelial cells were initially infected with RSV, and the infected epithelium was then inoculated with *P. aeruginosa*. After biofilm formation, antimicrobial peptides were added to mature biofilm cocultures for 1 h, and the remaining viable bacteria were quantified. While LL-37 was unable to disrupt *P. aeruginosa* biofilms under these conditions, WLBU2 was still effective, reducing CFU of *P. aeruginosa* during RSV coinfection by approximately 10-fold (Fig. 4B). Given the short treatment time and the extraordinary antimicrobial resistance of biofilms formed on biotic surfaces (23, 24), which is compounded during RSV coinfection (Fig. 4A), this result represents a significant effect compared to currently available antibiotics. To visualize the effects of WLBU2 on *P. aeruginosa* biofilms during RSV infection of airway epithelial cells, we next infected a confluent monolayer of bronchial epithelial cells with red fluorescent protein (RFP)-producing RSV and followed biofilm development of GFP-producing *P. aeruginosa* in a perfusion chamber by fluorescence microscopy. WLBU2 was able to reduce biomass by approximately 70% compared to treatment with vehicle (Fig. 4C).

While previous studies suggest WLBU2 can alter inflammatory responses of the airway epithelium (17), endogenous antimicrobial peptide expression was not altered during treatment of RSV-infected polarized bronchial epithelial cells with WLBU2 (see Fig. S2A in the supplemental material), indicating that WLBU2 is not acting indirectly on *P. aeruginosa* via induction of endogenous antimicrobial peptides. Cytokine and chemokine gene expression was also not significantly altered by WLBU2 treatment (see Fig. S2B). Along with the ability of WLBU2 to kill planktonic bacteria (18) and disrupt biofilms grown on abiotic surfaces (Fig. 1B and Fig. 2), these results are consistent with the conclusion that the antibacterial and antibiofilm effects of WLBU2 are direct rather than an inducible property of the airway epithelium.

10.1128/mSphere.00083-16.2 Figure S2  WLBU2 treatment does not alter endogenous antimicrobial peptide, chemokine/cytokine, or antiviral effector induction during viral infection. (A) Endogenous antimicrobial peptide gene expression in polarized bronchial epithelial cells infected with RSV after 5 h of apical treatment with 50 µM WLBU2. Fold change in gene expression (GAPDH normalized) is shown for RSV-infected cells treated with WLBU2 compared to those treated with vehicle. Induction of antimicrobial peptide genes during viral infection was not altered more than 3-fold by WLBU2 treatment. (B) Chemokine and cytokine gene expression in polarized bronchial epithelial cells infected with RSV after 5 h of apical treatment with 50 µM WLBU2. Fold change in gene expression (GAPDH normalized) is shown for RSV-infected cells treated with WLBU2 compared to those treated with vehicle. Induction of chemokine and cytokine gene expression was not altered more than 3-fold by WLBU2 treatment. (C) Interferon lambda 1 (IFN-λ1) and downstream antiviral interferon-stimulated gene (ISG) expression in polarized bronchial epithelial cells infected with RSV after 5 h of apical treatment with 50 µM WLBU2. Fold change in gene expression (GAPDH normalized) is shown for RSV-infected cells treated with WLBU2 compared to those treated with vehicle. Induction of antiviral signaling genes during viral infection was not altered more than 3-fold by WLBU2 treatment. Data are means from three independent experiments. Error bars indicate standard deviations. Download Figure S2, TIF file, 0.1 MB. Copyright © 2016 Melvin et al. 2016 Melvin et al. This content is distributed under the terms of the Creative Commons Attribution 4.0 International license.

### WLBU2 inhibits viral infectivity.

**FIG 5:**
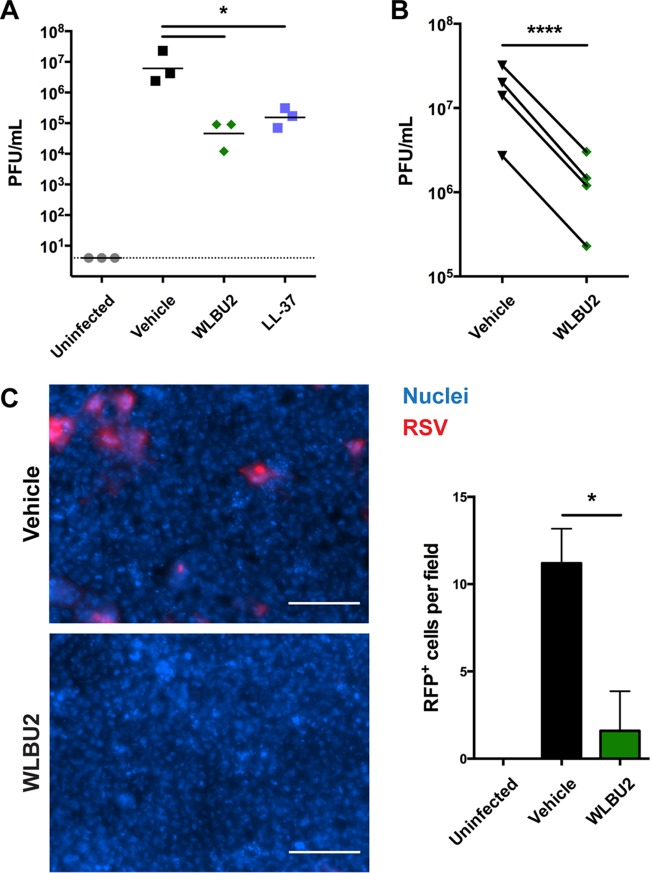
WLBU2 inhibits RSV infectivity. (A) Number of infectious particles in airway surface liquid of RSV-infected polarized bronchial epithelial cells after 5 h of apical treatment with 50 µM WLBU2 or LL-37. Dotted line indicates limit of detection. (B) Number of infectious particles after treatment with 50 µM WLBU2. RSV was inoculated in the presence of vehicle or 50 µM WLBU2. (C) Representative images and quantification of bronchial epithelial cells after 48 h postinfection with RFP-producing RSV. RSV was inoculated at an MOI of 5 in the presence of vehicle or 50 µM WLBU2. Bronchial epithelial cell nuclei are in blue, and RSV is in red. Scale bars, 100 µm. Data are means from three or more independent experiments. Error bars indicate standard deviations. *, *P* < 0.05; ****, *P* < 0.0001.

As antimicrobial peptides have been observed to be active against enveloped viruses (26), we investigated whether WLBU2 also might be active against the viral pathogen in our coinfection model. When polarized bronchial epithelial cells infected with RSV for 72 h were subsequently treated apically with WLBU2, the number of PFU in the airway surface liquid was reduced by over 150-fold (Fig. 5A). Similar to previous studies (27), LL-37 reduced the number of infective RSV particles about 50-fold. As viral transcription was not significantly altered by WLBU2 or LL-37 treatment (Fig. 4B), and subsequent antiviral interferon signaling was not significantly affected by addition of WLBU2 (see Fig. S2C in the supplemental material), this finding indicated that WLBU2 had negligible effects on the ability of the airway cells to control ongoing viral infection and that the antiviral effects of WLBU2 were likely due to direct inactivation of RSV rather than induction of a cellular antiviral response. To further investigate whether this effect was a direct action of WLBU2 on RSV or an indirect action on the airway cells releasing the virus, we coincubated WLBU2 with purified RSV prior to measuring infectious virus particles using a plaque assay. Infectivity was reduced over 10-fold (Fig. 5B), suggesting that WLBU2 directly interacts with RSV to inhibit infection. This result was supported by infection of polarized bronchial epithelial cells with an RFP-producing RSV, where coincubation of purified RSV with WLBU2 again reduced infectivity of the virus by approximately 85% (Fig. 5C).

Rising antibiotic resistance around the world poses a major threat to public health. As chronic infections characterized by the development of biofilms are particularly difficult to treat, antimicrobial agents capable of disrupting biofilms would be extremely valuable. WLBU2 is a unique antimicrobial peptide, as it is active against a broad range of highly resistant bacterial isolates (14, 18) and maintains activity under diverse physiological conditions (15, 16, 19). Here, we show the potential for engineered antimicrobial peptides to disrupt difficult-to-treat biofilms developed in the context of the airway epithelium, a challenging therapeutic environment.

**FIG 6:**
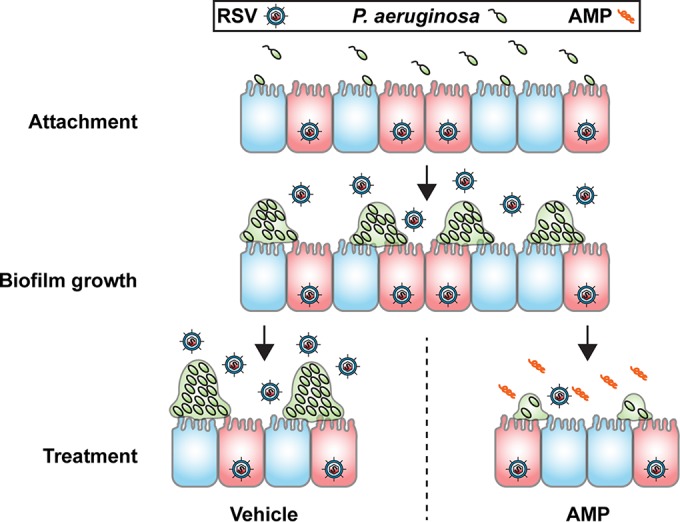
Model of *P. aeruginosa*-RSV coinfection of bronchial epithelium and treatment with antimicrobial peptides (AMP). *P. aeruginosa* first attaches to bronchial epithelial cells infected with RSV. These attached bacteria form biofilms on the RSV-infected epithelium. Lack of treatment results in robust biofilm proliferation and release of infectious viral particles in the airway lumen. Treatment with WLBU2 results in disruption of biofilms, reducing both the number of biofilms and the size of the biofilms that remain after 1 h of treatment, and reduction of infectious virus particles in the airway lumen.

Polymicrobial infections can be difficult to treat due to synergistic pathogenic interactions (28), which can be compounded by reduced antimicrobial susceptibility (11). Nevertheless, WLBU2 demonstrates the potential for simultaneous treatment of both a viral pathogen and a bacterial pathogen during a synergistic coinfection (Fig. 6), providing a single-molecule combination therapy. These findings highlight the therapeutic potential of engineered antimicrobial peptides for use in the treatment of especially recalcitrant infections, such as those in the lungs of CF patients.

## MATERIALS AND METHODS

### Peptide synthesis.

WLBU2 (RRWVRRVRRWVRRVVRVVRRWVRR) and LL-37 (LLGDFFRKSKEKIGKEFKRIVQRIKDFLRNLVPRTES) were synthesized using standard 9-fluorenylmethoxy carbonyl (Fmoc) synthesis protocols, purified by reverse-phase high-performance liquid chromatography (HPLC), and quantified by ninhydrin assay as previously described (14). Peptides were stored at −20°C as a 1 mM solution in phosphate-buffered saline (PBS). Vehicle treatments were performed using PBS.

### Strains and growth conditions.

*Pseudomonas aeruginosa* strain PAO1 containing a plasmid constitutively expressing the gene (*gfp*) encoding green fluorescent protein (23), *Staphylococcus aureus* strain USA100 containing a plasmid constitutively expressing *gfp* (29), and respiratory syncytial virus (RSV) line A2 and a variant constitutively expressing the gene (*rfp*) encoding red fluorescent protein were used in this study. *P. aeruginosa* was cultured in LB broth (Sigma), and *S. aureus* was cultured in tryptic soy broth with 10 µg/ml chloramphenicol (Sigma) overnight at 37°C prior to use in coculture experiments. Bacterial cultures were washed in minimum essential medium (MEM [Gibco]) supplemented with 2 mM l-glutamine (Corning) prior to inoculation. Immortalized human bronchial epithelial cells from a ΔF508 homozygous cystic fibrosis patient (CFBE41o^−^) stably expressing wild-type *CFTR* (CFBE-wt) (23) were cultured in MEM (Gibco) supplemented with 2 mM l-glutamine, 5 U/ml penicillin, and 5 µg/ml streptomycin (Sigma), 0.5 µg/ml Plasmocin prophylactic (InvivoGen), and 10% fetal bovine serum (FBS [Gemini Bio-Products]). CFBE-wt cells were obtained from J. P. Clancy at Cincinnati Children’s Hospital. Immortalized murine fibroblast cells from a STAT1^−/−^ mouse (NY3.2) (30) were cultured in MEM supplemented with 5 U/ml penicillin, 5 µg/ml streptomycin, and 5% FBS. NY3.2 cells were obtained from J. E. Durbin at Rutgers, New Jersey Medical School. CFBE-wt and NY3.2 cells are not on the commonly misidentified list, and cells were tested monthly for mycoplasma using a Southern Biotech mycoplasma detection kit.

### Static coculture biofilm disruption.

CFBE-wt cells were polarized on Transwell permeable supports (Costar) at the air-liquid interface for 7 days prior to use (5). For coinfection assays, cells were infected with RSV diluted in MEM supplemented with 2 mM l-glutamine to a multiplicity of infection (MOI) of 1 for 72 h prior to inoculation with *P. aeruginosa* at an MOI of 25. *P. aeruginosa* cells were allowed to attach for 1 h before unattached bacteria were removed, and the apical medium was replaced with MEM supplemented with 2 mM l-glutamine and 0.4% l-arginine (Sigma-Aldrich). After 2 h of biofilm development, apical medium was replaced with MEM supplemented with 2 mM l-glutamine containing vehicle, 50 µM WLBU2, or 50 µM LL-37 (Gibco). After 1 h of treatment, apical medium was removed, and cells were washed with MEM. MEM containing 0.1% Triton X-100 (Bio-Rad) was used to disrupt epithelial cells and biofilms. Bacteria were serially diluted and plated on LB agar (Fluka) to determine CFU. *S. aureus* biofilm disruption was performed similarly, with minor alterations. Bacterial attachment was allowed to proceed for 1 h and biofilm development for 4 h, with 2 h of treatment. *S. aureus* CFU were enumerated on tryptic soy agar. Tobramycin and ciprofloxacin susceptibility was also assessed using the static coculture system. CFU for each Transwell were enumerated in duplicate and averaged. Treatment conditions were applied to duplicate Transwells, and the CFU recovered were averaged.

### Flow coculture biofilm disruption.

CFBE-wt cells were seeded at a high density and grown as a confluent monolayer on glass coverslips (Fisher Scientific) for 7 days prior to use (23), at which point the cells form tight junctions that are capable of excluding bacterial transit to the bottom of the monolayer. For coinfection assays, cells were infected with RSV constitutively expressing red fluorescent protein (RFP) diluted in MEM to an MOI of 1 for 6 h. Cells were returned to growth medium for 18 h prior to inoculation with *P. aeruginosa*. Bacteria were inoculated at an MOI of 25. Bacteria were allowed to attach for 2 h before unattached bacteria were removed by perfusion with MEM supplemented with 2 mM l-glutamine at 20 ml/h (23). After 2 h of biofilm development, 10 random z-stacks were collected from each chamber using a Nikon Ti-inverted microscope to obtain “pretreatment” images. MEM supplemented with 2 mM l-glutamine containing vehicle or 10 µM WLBU2 was then perfused through the chamber. After 1 h of treatment, 10 random z-stacks were collected from each chamber using a Nikon Ti-inverted microscope to obtain “posttreatment” images. Biofilm biomass was quantified using Nikon Elements software (5).

### Viral infectivity determination.

For static coculture biofilm disruption assays, viral infection, antimicrobial peptide gene expression, cytokine and chemokine gene expression, and interferon signaling gene expression were evaluated via RNA isolation with the RNeasy minikit (Qiagen), cDNA synthesis was achieved with the iScript cDNA synthesis kit (Bio-Rad), quantitative PCR (qPCR) was performed using iQ SYBR green supermix (Promega) with the primers listed in Table S1 in the supplemental material, and fold change was calculated after normalization to glyceraldehyde-3-phosphate dehydrogenase gene (*GAPDH*) expression. RSV-infected cells were treated with vehicle or 50 µM WLBU2 as described for the biofilm assays prior to RNA isolation.

10.1128/mSphere.00083-16.3 Table S1  Primers used for RT-qPCR. Download Table S1, TIF file, 0.4 MB. Copyright © 2016 Melvin et al. 2016 Melvin et al. This content is distributed under the terms of the Creative Commons Attribution 4.0 International license.

The remaining RSV infectious particles, from apical secretions of RSV-infected polarized CFBE-wt cells or purified RSV, after treatment with vehicle, 50 µM WLBU2, or 50 µM LL-37 diluted in Dulbecco’s modified Eagle’s medium (DMEM) plus GlutaMAX supplemented with 1% FBS and 25 mM HEPES were quantified by plaque assay. Briefly, polystyrene plates were coated with polyethylenimine (Sigma) and seeded to confluence with NY3.2 cells. Viral attachment was allowed to occur in DMEM plus GlutaMAX supplemented with 1% FBS and 25 mM HEPES for 2 h before cells were covered with a methylcellulose-polyethylene glycol overlay. After 48 h of infection, cells were fixed in 10% buffered formalin phosphate (Fisher Scientific). Plaque detection was achieved with an anti-RSV antibody (Meridian Life Science, Inc.) and an alkaline phosphatase (AP)-conjugated anti-goat antibody (Santa Cruz Biotechnology). Plaques were developed with SIGMA*FAST* BCIP/NBT (5-bromo-4-chloro-3-indolylphosphate–nitroblue tetrazolium [Sigma]). Four separate preparations of RSV were tested.

RSV infectivity after treatment of virus with vehicle or 50 µM WLBU2 diluted in MEM was visualized by staining polarized CFBE-wt cells with Hoechst 33342 (Invitrogen), infecting cells with RFP-RSV at an MOI of 5 for 48 h, fixing with 4% paraformaldehyde (Alfa Aesar), and mounting filters with ProLong Gold antifade mountant (Thermo, Fisher Scientific). Images were obtained using a Nikon Ti-inverted microscope. Five random fields were collected, and the number of RFP-positive cells was counted.

### TEER measurement.

Transepithelial electrical resistance (TEER) was measured hourly for 3 h on air-liquid interface differentiated CFBE-wt airway epithelial cells treated with vehicle, 10 or 50 µM WLBU2, or 10 or 50 µM LL-37 diluted in MEM using an Ag/AgCl electrode (EVOM^2^ meter; World Precision Instruments).

### LDH release.

Lactate dehydrogenase (LDH) release was measured on air-liquid interface differentiated CFBE-wt airway epithelial cells treated for 5 h with vehicle or 20 or 50 µM WLBU2 diluted in MEM. Measurements were performed on control uninfected cells and cells that were infected with *P. aeruginosa* for 1 h prior to peptide or vehicle treatment, using the CytoTox 96 nonradioactive cytotoxicity assay (Promega). *P. aeruginosa* infection was performed as described above.

### Abiotic surface biofilm disruption.

For propidium iodide (PI) uptake imaging, *P. aeruginosa* cells were grown in glass-bottom dishes (MatTek Corporation) in MEM for 24 h. Dishes were washed with MEM, and bacteria were exposed to 125 ng/ml propidium iodide (Sigma-Aldrich) and 50 µM WLBU2, 50 µM LL-37, 1,000 µg/ml tobramycin, or vehicle diluted in MEM for 1 h. Images were obtained using a Nikon Ti-inverted microscope. Five random fields were collected at each time point, and the number of PI-positive bacteria was counted using Nikon Elements software.

For biomass measurements, *P. aeruginosa* cells were grown in a vinyl microtiter plate in MEM for 24 h in a humidified incubator at 37°C and 5% CO_2_ prior to treatment with various concentrations of WLBU2 diluted in MEM for 1 h. Biomass was stained with crystal violet (41% crystal violet, 12% ethanol, 47% H_2_O) and quantified by solubilization in 30% acetic acid and measurement of absorbance at 550 nm on a SpectraMax M2 microplate reader (Molecular Devices) (31). Treatments were performed in triplicate, and absorbance values were averaged.

### Statistical analysis

Data were plotted and statistical analyses were performed using Prism version 6.0 software (GraphPad Software, Inc.). For all conditions analyzed, experiments were performed on at least 3 separate days. Statistical significance was determined using analysis of variance (ANOVA), correcting for multiple comparisons.
