# Supplementary figures and images for: Simultaneous Antibiofilm and Antiviral Activities of an Engineered Antimicrobial Peptide during Virus-Bacterium Coinfection
Source: mSphere. 2016 May 4;1(3):e00083-16. doi: 10.1128/mSphere.00083-16 (PMC4888888; doi:10.1128/mSphere.00083-16)

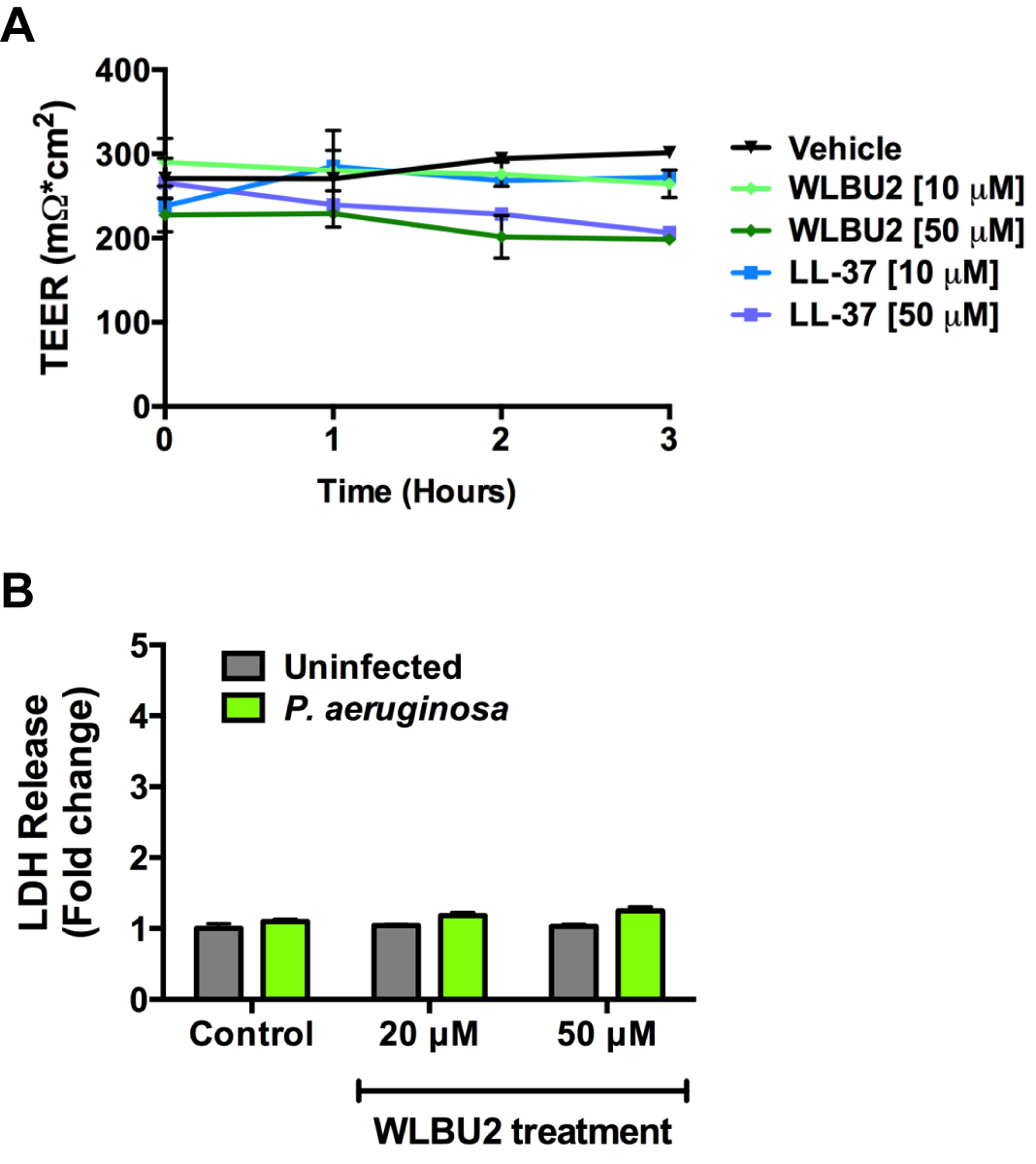

Supplement: Figure S1 [file sph003162080sf1.tif]

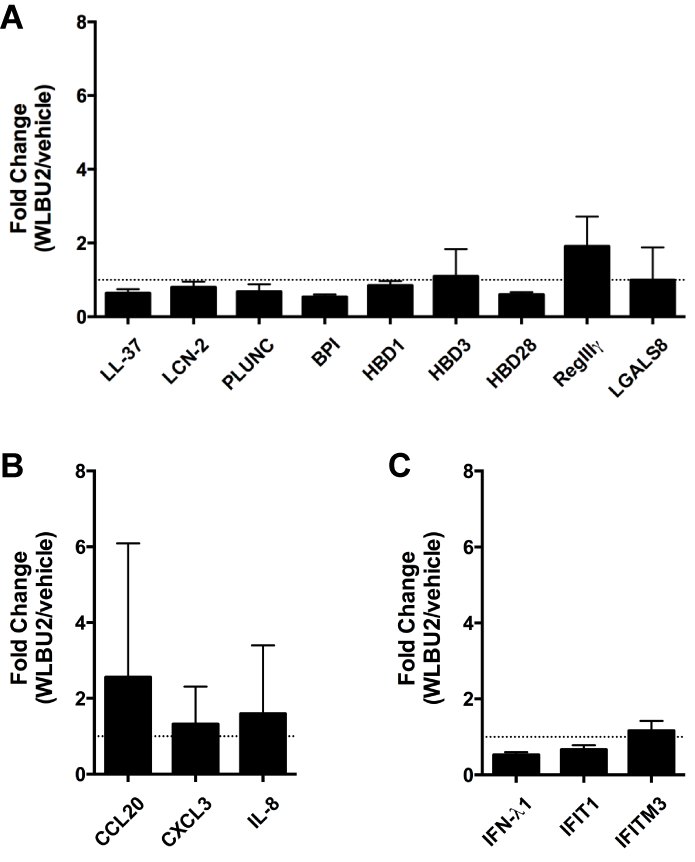

Supplement: Figure S2 [file sph003162080sf2.tif]

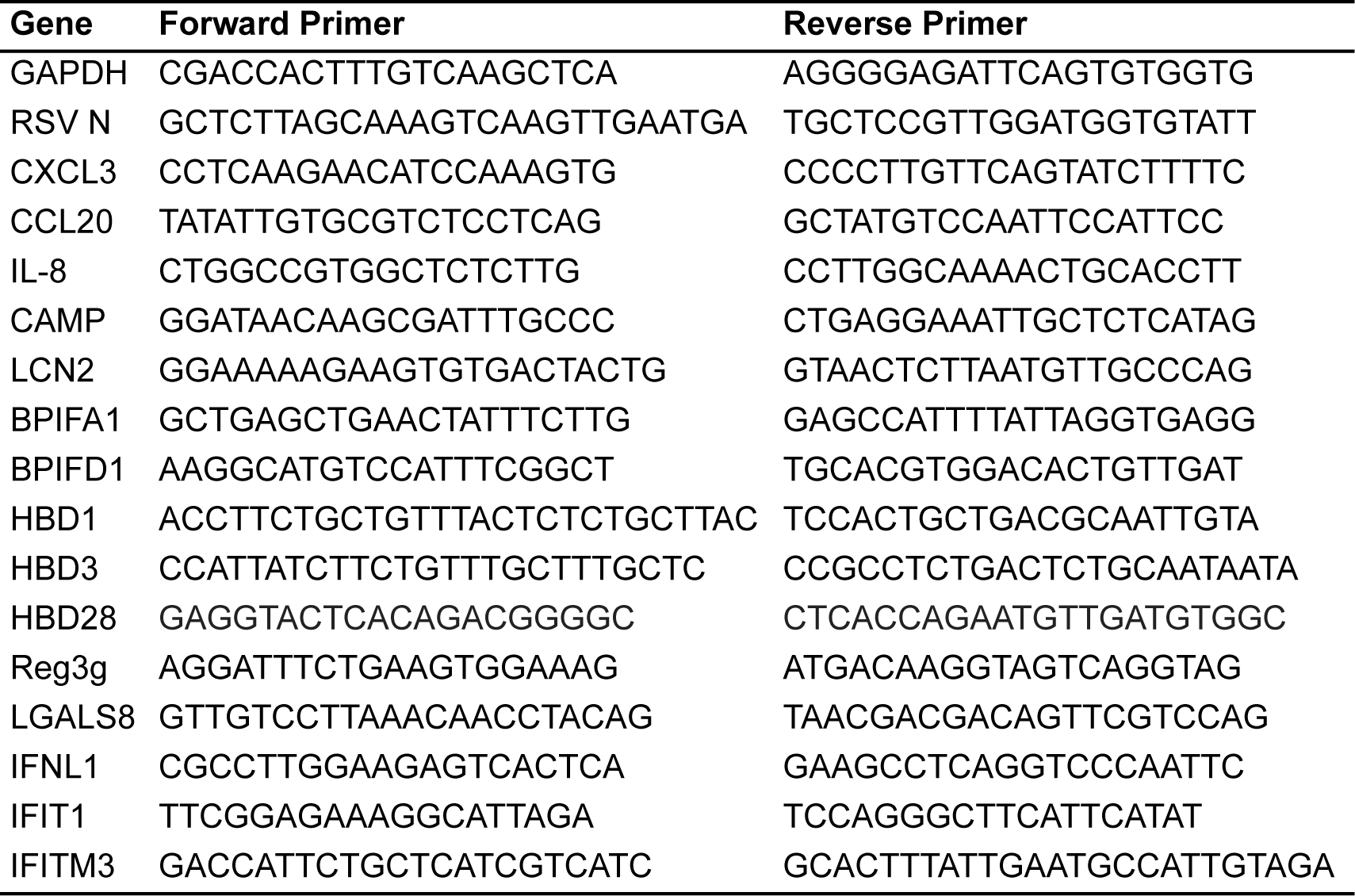

Supplement: Table S1 [file sph003162080st3.tif]
